# Can the FAST and ROSIER adult stroke recognition tools be applied to confirmed childhood arterial ischemic stroke?

**DOI:** 10.1186/1471-2431-11-93

**Published:** 2011-10-21

**Authors:** Adriana Yock-Corrales, Franz E Babl, Ian T Mosley, Mark T Mackay

**Affiliations:** 1 Emergency Department, Royal Children's Hospital, Melbourne, Australia; 2 Murdoch Children's Research Institute, Melbourne, Australia; 3 University of Melbourne, Melbourne, Australia; 4 National Stroke Research Institute, Melbourne, Australia; 5 Monash University, Melbourne, Australia; 6 Department of Neurology, Royal Children's Hospital, Melbourne, Australia

**Keywords:** Stroke, stroke recognition tools, FAST, ROSIER, child, emergency department

## Abstract

**Background:**

Stroke recognition tools have been shown to improve diagnostic accuracy in adults. Development of a similar tool in children is needed to reduce lag time to diagnosis. A critical first step is to determine whether adult stoke scales can be applied in childhood stroke.

Our objective was to assess the applicability of adult stroke scales in childhood arterial ischemic stroke (AIS)

**Methods:**

Children aged 1 month to < 18 years with radiologically confirmed acute AIS who presented to a tertiary emergency department (ED) (2003 to 2008) were identified retrospectively. Signs, symptoms, risk factors and initial management were extracted. Two adult stroke recognition tools; ROSIER (Recognition of Stroke in the Emergency Room) and FAST (Face Arm Speech Test) scales were applied retrospectively to all patients to determine test sensitivity.

**Results:**

47 children with AIS were identified. 34 had anterior, 12 had posterior and 1 child had anterior and posterior circulation infarcts. Median age was 9 years and 51% were male. Median time from symptom onset to ED presentation was 21 hours but one third of children presented within 6 hours. The most common presenting stroke symptoms were arm (63%), face (62%), leg weakness (57%), speech disturbance (46%) and headache (46%). The most common signs were arm (61%), face (70%) or leg weakness (57%) and dysarthria (34%). 36 (78%) of children had at least one positive variable on FAST and 38 (81%) had a positive score of ≥1 on the ROSIER scale. Positive scores were less likely in children with posterior circulation stroke.

**Conclusion:**

The presenting features of pediatric stroke appear similar to adult strokes. Two adult stroke recognition tools have fair to good sensitivity in radiologically confirmed childhood AIS but require further development and modification. Specificity of the tools also needs to be determined in a prospective cohort of children with stroke and non-stroke brain attacks.

## Background

Recent studies have confirmed that diagnostic delays, are a key factor impacting on access to acute stroke interventions in children [1-5] Stroke symptoms in children are often attributed to other problems such as migraine, encephalitis, tumors or Todd's paralysis due to limited knowledge about the signs and symptoms of stroke amongst primary care physicians, pediatricians and emergency physicians [5].

Clinical stroke recognition tools have been developed to improve recognition of stroke symptoms in adults. Instruments including the Face Arm Speech Test (FAST), the Cincinnati Pre-Hospital Stroke Scale (CPSS) and the Los Angeles Pre-Hospital Stroke Screen (LAPSS) [6-10] have been shown to improve paramedic diagnostic accuracy in identifying stroke. Use of the Recognition of Stroke in the Emergency Department (ROSIER) tool by emergency physicians has also been shown to have good sensitivity and specificity in differentiating between stroke and non-stroke [9].

Development of similar tools may decrease time to stroke diagnosis in children but it is unclear whether pediatric stroke has similar presenting characteristics to adult stroke. Our aim was to retrospectively describe the spectrum of symptoms and signs in children presenting to a tertiary emergency department (ED) with acute arterial ischemic stroke (AIS). We hypothesised that the presenting characteristics of childhood stroke in children are similar to those encountered in adults.

## Methods

### Study design

Retrospective consecutive case series of children aged 1 month to less than 18 years who presented to the Royal Children's Hospital, Melbourne (RCH) ED with (AIS) during the five year period between January 2003 and December 2008. The study was approved by the institutional hospital research ethics committee at Royal Children's Hospital with a waiver of consent for enrolled patients.

### Study setting

RCH is the tertiary pediatric referral centre for the state of Victoria, Australia, with a population of 5 million. Annual ED census is 67, 000 and hospital census is 280, 000 patients.

### Case identification, inclusion and exclusion criteria

Cases were ascertained from our institutional stroke registry, established in 2002. Patients identified were cross-referenced with a separate ED electronic hospital admission database to ensure all potential patients were included. Patients with perinatal arterial ischemic stroke, primary haemorrhagic stroke, subdural, extradural hemorrhage and cerebral sino-venous thrombosis were excluded from this study. Patients with incomplete medical records and patients that did not present first to the ED (e.g. direct admission to ICU or ward) were also excluded.

### Definitions

AIS was defined as acute neurological deficits lasting more than 24 hrs caused by cerebral ischemia, with neuroimaging showing parenchymal infarction, conforming to known arterial territories and corresponding to the clinical presentation [11]. Anterior circulation stroke was defined at infarction in the anterior and middle cerebral artery territories and posterior circulation stroke as infarction in the vertebrobasilar territories.

ED triage urgency was assessed using the national Australian triage scale (ATS) [12]. Patients with ATS category 1, 2, 3, 4 and 5 are to be seen immediately, within 10, 30, 60 and 120 minutes respectively. For young children a modified Glasgow Coma Scale was used [13].

A pediatric modification of the TOAST classification system was used to define stroke subtypes including sickle cell disease, cardiac embolism, cervical arterial dissection, Moyamoya disease, steno-occlusive cerebral arteriopathy, other determined etiology, multiple probable/possible etiologies and undetermined etiology. All patients with undetermined etiology had normal investigations including cerebrovascular imaging, echocardiogram, and prothrombotic studies [14,15].

### Study protocol

Data was abstracted from electronic and written ED records and from the medical record admission and progress notes. Laboratory and radiology reports were also reviewed. Data collected included demographics, age, timing of onset of symptoms and presenting symptoms and signs. Clinical variables were selected on the basis of symptoms or signs found to be useful for discriminating stroke from non-stroke at the bedside in adult studies [6,9,11,16]. Symptoms and signs were extracted based on the first ED physician's notes and the first neurologist's or neurosurgeon's notes in the ED and medical records. Portions of the data were abstracted by two investigators and entered twice.

Two adult stroke recognition tools were retrospectively applied to our population. The FAST prehospital stroke recognition tool has been shown to have high positive predictive value when used by paramedics, primary care physicians and emergency physicians. It has also been used by the National Stroke Foundation of Australia in community stroke awareness campaigns. FAST variables include facial palsy, arm weakness and speech impairment [6,10,17]. A positive result is defined as the presence of at least one variable.

The ROSIER scale was also retrospectively applied to our stroke population [9]. This tool has been developed for emergency physicians and it has also been demonstrated to be a simple, sensitive and specific tool for assisting non neurologists in the clinical assessment of potential stroke patients. The ROSIER is a seven item scoring system ranging from -2 to +5 that includes the following neurological signs and symptoms: loss of consciousness or syncope, seizure activity, acute onset asymmetrical facial weakness, asymmetrical arm weakness, asymmetrical leg weakness, speech disturbance and visual defect. The first two items are more discriminatory for non stroke diagnosis and are given a score of -1 and the remainder are more discriminatory for stroke and are given a score of +1. The optimum cut off point for stroke is a total score of +1 or above.

### Data analysis

Clinical parameters of AIS were analysed descriptively. Data were entered in an Epidata database and analysed using Stata (version 10.0 Stata Corporation, Texas, USA).

## Results

A search of the RCH stroke registry identified 55 patients during the 5 year study period. A total of 47 patients were included in the study; 34 (72%) had anterior circulation stroke, 12 (26%) had posterior circulation stroke and one child (2%) had involvement of both. (Table 1). Eight patients were excluded: 5 had incomplete documentation and 3 had a transient ischaemic attacks without radiological evidence of parenchymal infarction.

**Table 1:** Demographic Characteristics and Clinical Findings of Children with Acute Ischemic Stroke (AIS)

*Variable*	*AIS*			*Anterior Circulation* *n = 34*		*Posterior Circulation* *n = 12*		*Both Circulations* *n = 1*	
**Demographics**	**N**	**n**	**%**	**n**	**%**	**n**	**%**	**n**	**%**
**Age, mean**	47	7	SD 5.2	6.3	SD 5.2	9.5	SD 5.1	3.6	-
**Age < 2 years**	47	9	19	8	23	1	8	-	-
**Male sex**		24	51	17	50	6	50	1	100
Triage category*									
**1+2**	47	12	25	9	26.5	3	25	-	-
**3+4+5**	47	35	74	25	73.5	9	75	1	100
**Arrival by ambulance**	47	27	57	19	55	8	67	-	-
**Complaints**									
**Time presentation < 6 hrs**	47	15	32	14	41	1	8	-	-
**Well week before**	47	41	87	29	85	11	92	1	100
**Sudden onset**	47	39	83	31	91	8	67	1	100
**Woke from sleep**	47	10	21	6	18	4	33	-	-
**Worsening symptoms**	47	25	53	16	47	8	67	1	100
**Arm weakness**	47	30	63	25	73	5	42	-	-
**Leg weakness**	47	27	57	22	64	5	42	-	-
**Face weakness**	47	29	62	24	71	5	42	-	-
**Limb incoordination/ataxia**	45	11	23	7	21	4	33	-	-
**Speech disturbances**	45	22	46	16	47	6	50	-	-
**Visual disturbances**	47	7	14	1	3	6	50	-	-
**Arm numbness**	47	3	6	2	6	1	8	-	-
**Leg numbness**	47	2	4	1	3	1	8	-	-
**Face numbness**	47	4	8	3	9	1	8.3	-	-
**Vomits**	47	9	19	6	17	3	25	-	-
**Headache**	45	22	46	12	35	9	75	1	100
**Altered mental status**	47	9	19	6	17	2	17	1	100
**Seizure**	47	8	17	6	17	2	17	-	-
No lateralizing symptoms**	47	18	38	10	29	7	58	1	100
**Neurological Examination**									
GCS***									
**≥14**	47	40	85	29	85	11	92	-	-
**> 8- < 14**	47	7	15	5	15	1	8	1	100
**≤ 8**	47	-	-	-	-	-	-	-	-
**Arm weakness**	47	29	61	25	73	4	33	-	-
**Leg weakness**	47	27	57	23	67	4	33	-	-
**Face asymmetry**	47	33	70	28	82	5	42	-	-
**Dysarthria**	44	15	34	11	32	4	33	-	-
**Dysphasia**	44	6	14	6	17	-	-	-	-
**Visual defect/eye movement abnormality**	37	7	19	3	9	4	33	-	-
**Sensory loss**	47	12	25	10	29	2	17	-	-
**Ataxia**	47	5	10	2	6	3	25	-	-

*Triage category (see methods)

***GCS Glasgow Coma Scale (see methods)

**All patients with no lateralising motor or sensory symptoms had other symptoms including visual disturbances, headache/vomiting, altered concious state, and seizures.

Patients ranged in age from 4 months to 16 years with a median age of 7 years. Fifty-four percent arrived by ambulance and thirty-eight percent of the patients brought by their parents arrived by private car. High triage categories 1 and 2 were assigned to only 25% of AIS patients. (Table 1) Forty-seven percent of patients had prior relevant medical history including history of migraine/headaches (7), head/neck trauma (5), previous stroke (3), chickenpox (2), cardiac disease (2) and other diagnosis (4). Median time from onset of symptoms to the ED presentation was 21 hours (IQR 5.7-48).

### Clinical Data

Table 1 shows presenting complaints and neurological examination findings. The majority (83%) of patients presented with sudden onset of symptoms and one third presented within 6 hours of stroke onset. The most common presenting symptoms were arm (63%), facial (62%) or leg weakness (57%), speech disturbances (46%) and headache (46%). Focal arm face or leg weakness were more common with anterior circulation stroke and visual disturbance or headache were more common with posterior circulation stroke. Most patients (85%) had a GCS of ≥ 14. The most common neurological signs were facial (70%), arm (61%) or leg weakness (57%) and slurred speech (34%). Once again focal arm, face or leg weakness were more common with anterior circulation stroke and visual disturbance or ataxia were more common with posterior circulation stroke

Patients were classified according to the Pediatric Stroke Classification [14,15] (Table 2).

**Table 2:** Etiology of Arterial Ischemic Stroke (AIS) [14,15]

	AIS N = 47	
Pediatric Stroke Classification*	**n**	**%**
**Steno-occlusive arteriopathy**	17	36
**Undetermined**	18	38
**Moyamoya syndrome**	6	13
**Arterial Dissection**	4	8
**Cardioembolic**	1	2
Probable/Possible aetiology*	1	2

*Prothrombotic state without additional risk factors

### Stroke Scales

The FAST and ROSIER stroke recognition tools were applied retrospectively. As shown in Table 3 the majority of the patients had facial weakness (66%) or arm weakness (59%). Thirty-six (76%) of the patients with AIS had at least one positive variable from the FAST scale but there were differences between anterior and posterior circulation strokes (88% compared to 50%).

**Table 3:** Application of FAST (Face Arm Speech Test) Stroke Scale to Children with Acute Ischemic Stroke (AIS)

*Variable*	*AIS* * N= 47*		*Anterior Circulation* * n= 34*		*Posterior Circulation* * n= 12*		*Both Circulations* * n= 1*	
**FAST**	**n**	**%**	**n**	**%**	**n**	**%**	**n**	**%**
**Face**	33	70	26	76	5	41.6	-	-
**Arm**	29	61	24	71	4	33.3	-	-
**Speech**	15	34	13	38	5	41.6	-	-
**At least 1 of above**	**36**	**76**	**30**	**88**	**6**	**50**	**-**	**-**

Thirty eight (81%) of children had a positive ROSIER with a total score of ≥+1 (Table 4). Positive variables are shown in Table 5 with asymmetrical arm weakness being the most common neurological sign of stroke. Vascular territory also influenced results with a positive ROSIER score in 85% of anterior and 75% of posterior circulation strokes.

**Table 4:** Application of ROSIER (Recognition of Stroke in the Emergency Department) Stroke Scale to Children with Acute Ischemic Stroke (AIS)

*Variable*	*AIS* * N = 47*	
**ROSIER**	**n**	**%**
-2	0	0
-1	3	6
0	6	13
**+1**	**8**	**17**
**+2**	**9**	**19**
**+3**	**11**	**23**
**+4**	**9**	**19**
**+5**	**1**	**2**

**Table 5:** Application of ROSIER (Recognition of Stroke in the Emergency Room) Stroke Scale to Children with Acute Ischemic Stroke (AIS)

	*AIS* * N = 47*		*Anterior Circulation* * n = 34*		*Posterior Circulation * *n = 12*		*Both Circulations* * n = 1*	
	**n**	**%**	**n**	**%**	**n**	**%**	**n**	**%**
**Loss of consciousness or syncope?**	0	0	0	0	0	0	0	0
**Seizure**	8	17	6	17	2	17	0	0
**I. Asymmetric facial weakness**	33	70	28	82	5	42	0	0
**II. Asymmetric arm weakness**	29	61	25	73	4	33	0	0
**III. Asymmetric leg weakness**	27	57	23	67	4	33	0	0
**IV. Speech disturbance**	15	34	11	32	4	33	0	0
**V. Visual defect**	4	10	2	6	2	17	0	0
**Positive ROSIER with cut off ≥ 1**	38	81	29	85	9	75	0	0

The ROSIER performed similarly in children aged less than 2 years when compared to older children. Seven of 9 had a positive score at a similar rate to the overall group. One of the 2 younger patients with ROSIER of < 1 had a posterior circulation stroke.

Three of 8 patients with seizures had a ROSIER of < 1, suggesting that ROSIER appears to be largely positive in these children due to other neurological signs and symptoms. When removing the seizure variable from the ROSIER score, the number of patients with a negative ROSIER remained unchanged at 9 children.

## Discussion

The aims of our study were to describe the spectrum of signs and symptoms of radiologically confirmed stroke in children presenting to a tertiary emergency department and more specifically to determine whether adult stroke recognition tools could be applied in childhood stroke. We found that face, arm or leg weakness and speech disturbance were the most common clinical signs of stroke and that two adult stroke recognition tools, FAST and ROSIER had fair to good sensitivity of 76% and 81% respectively for detection of stroke symptoms. Mode of presentation was influenced by vascular territory with lateralised limb weakness and sensory disturbance being more common in anterior circulation stroke and visual field defects, eye movement abnormalities and limb ataxia being more common in posterior circulation stroke. There was a corresponding difference in the sensitivity of the FAST and ROSIER tools, being lower in posterior circulation stroke.

Overall, this study shows that childhood AIS has similar clinical features to adult stroke [16]. In adults the most common presenting signs of stroke are arm weakness (68-81%), leg weakness (54-73%), facial weakness (45-59%), speech disturbance (45-59%) and sensory loss (36-49%) [9,16,18] largely similar to our pediatric stroke data. There is limited data on the presenting clinical features of stroke in children and our study provides in depth analysis of all neurological signs and symptoms at presentation. Previous studies have described clinical findings in terms of focal sign [4], motor deficits [2,3] or hemiparesis [19-21]. Some studies have described speech and visual field deficits [22] but sensory deficits, cranial nerve or cerebellar signs have only been reported in one study [2]. In contrast seizures at presentation have been more consistently reported in the pediatric literature, occurring in 11-28% of children at the time of initial presentation which is consistent with our findings.

Paramedics and emergency physicians are usually the first health professionals to assess patients with possible stroke. Both play important roles in expediting triage and assessment of patients and facilitating appropriate diagnostic imaging. Pre-hospital tools are used by ambulance paramedics to recognise potential stroke. The Face Arm Speech test (FAST) [6,10], has been shown to have high positive predictive value when used by paramedics (78%), primary care physicians (77%) and emergency physicians (71%) and is widely used in some countries including in Australia. It has a diagnostic sensitivity of 79% when used by paramedics. Our data suggests that the FAST has similar sensitivity to adults when applied to a group of children with confirmed childhood ischemic stroke.

Clinical diagnostic accuracy of ED physicians varies from 22-96% [9]. Therefore clinical tools have been developed for adults presenting with brain attack symptoms to assist clinicians in distinguishing between stroke and non stroke mimics. The *Recognition of Stroke in the Emergency Room *(ROSIER) scale has been developed for use by emergency physicians [9]. In order of discriminatory value acute onset of symptoms, arm weakness, leg weakness, speech disturbance or facial weakness predict stroke and seizures, confusion or loss of consciousness predict non stroke diagnosis. Sensory symptoms, vertigo, dizziness and HA are non discriminatory between stroke and mimic. ROSIER has a sensitivity of 91%, better than the prehospital tools such as FAST, CPSS and LAPSS [6-10] and a specificity of 92%, similar to or better than the prehospital tools. The ROSIER had better sensitivity than the FAST in our pediatric patients with a positive result seen in 81% of cases but it is still less than adults.

Signs and symptoms of stroke may be more difficult to identify in very young children but the proportion of children under age two with a positive ROSIER was similar to that of the group as a whole. Seizures are a predictor of non stroke diagnosis in adults with the ROSIER tool but they are a relatively common occurrence in childhood stroke, reported in 11-28% of cases [2,4,19]. The ROSIER remained positive in more than half of the children with seizures in our study, due to presence of other neurological signs predictive of stroke. However, firm conclusions cannot be drawn due to the small numbers in these subgroups and evaluation in a larger population of children is warranted.

Stroke topography has been shown to affect applicability of these tools in adults. None of the tools assess visual field defects, disorders of perception or balance so they are insensitive to posterior circulation. For example the CPSS has a sensitivity of 88% for anterior circulation strokes but only 29% for posterior circulation strokes [8]. The ROSIER incorrectly diagnosed stroke or mimic in 11% of cases and the majority of false negative cases were posterior circulation events [9]. It is possible that similar problems may be encountered in the pediatric population because approximately one quarter of our pediatric strokes involved the posterior circulation, at a similar frequency to adults [23].

There is a need to develop and validate appropriate pediatric bedside tools in the emergency department to improve the diagnostic accuracy in detection of childhood stroke. Our data indicate that adult stroke tools are a reasonable starting point but high false negative rates of 24% for the FAST and 19% for the ROSIER tool suggest they require further development and modification if they are to be useful in the pediatric emergency department.

The limitations of this retrospective study were that some symptoms or signs may not have been documented by the assessing physician or that those recorded may not have reflected the findings at initial presentation as the medical records included notes of the senior neurologist under whom all patients were admitted. Ideally the sensitivity of the scales would have been assessed for use by ED staff and neurologists separately to determine the utility of the scales when used by front line ED staff. These differences, however, could not be determined due to the retrospective methodology of this study. Again, due to the retrospective nature of the study, we accepted broadly interpreted descriptions in the medical records for items such as "sudden onset" or "woke from sleep" which were adapted from, though not further defined in an adult study by Hand et al [16]. Adult stroke scales were also designed to be applied prospectively. The study was conducted in a tertiary paediatric hospital so the results may not be generalisable to the broader paediatric population.

## Conclusions

Face, arm, or leg weakness and speech disturbances are good discriminatory signs of stroke in adults. We found that they are also common presenting features of stroke in children. This preliminary data suggests that stroke recognition tools like the FAST and ROSIER may be applicable to children in the prehospital and emergency settings. However they need to be tested by ED front line staff, in a prospective cohort study comparing stroke to non stroke brain attacks, to better define differences in presenting feature between these groups. This will also allow determination of specificity as well as negative and positive predictive values of stroke recognition tools in children. The effect of factors such as stroke topography, age and etiology also need to be determined.

## Abbreviations

AIS: acute ischemic stroke; ROSIER: Recognition of Stroke in the Emergency Room; FAST: Face Arm Speech Time; CPPS: Cincinnati Pre-Hospital Stroke Scale; LAPSS: Los Angeles Pre-Hospital Stroke Screen; LOC: loss of consciousness; GCS: Glasgow Coma Scale

## Conflict of interests

The authors declare that they have no competing interests.

## Authors' contributions

All authors contributed to the design, methodology, ethics application, data interpretation and drafting of the study. AYC was responsible for primary data acquisition. All authors have read and approved the final manuscript.

## Pre-publication history

The pre-publication history for this paper can be accessed here:

http://www.biomedcentral.com/1471-2431/11/93/prepub
